# Did the 2018 megadrought change the partitioning of growth between tree sizes and species? A Swiss case‐study

**DOI:** 10.1111/plb.13380

**Published:** 2021-12-22

**Authors:** A. K. Bose, B. Rohner, A. Bottero, M. Ferretti, D. I. Forrester

**Affiliations:** ^1^ Swiss Federal Institute for Forest, Snow and Landscape Research WSL Birmensdorf Switzerland; ^2^ Forestry and Wood Technology Discipline Khulna University Khulna Bangladesh; ^3^ SwissForestLab Birmensdorf Switzerland; ^4^ WSL Institute for Snow and Avalanche Research SLF Davos Dorf Switzerland; ^5^ Climate Change, Extremes and Natural Hazards in Alpine Regions Research Center (CERC) Davos Dorf Switzerland

**Keywords:** Climate change impacts, drought sensitivity, European beech, extreme droughts, growth dominance, tree size‐growth relationship

## Abstract

- By killing or weakening trees, drought could change the partitioning of growth between tree sizes or species, thereby altering stand structure. Growth partitioning, often quantified using the growth dominance coefficient (*DC*) or the shape of tree size *versus* growth relationships (*SGR*), indicates the relative contribution of differently sized trees to the total stand growth. Changes in growth partitioning due to droughts are rarely examined but provide valuable information that links tree‐ and stand‐level responses to droughts.
- The objective of this study was to test whether the 2018 European megadrought altered the growth partitioning among tree sizes and species. For this purpose, we first evaluated whether *DC* or *SGR* can be calculated from small sample sizes of trees typical of individual forest inventory plots.
- *DC*, and particularly *SGR*, were sensitive to sample size, forest type (even‐aged and uneven‐aged), target variable (tree diameter, basal area or stem mass) and range of tree sizes within the sample. *SGR* could therefore not be used for our analyses. We found no differences in *DC* prior to and during the 2018 drought. However, when considering only beech (*Fagus sylvatica*)‐dominated stands, *DC* was lower during post‐drought years than during the 2018 drought.
- The growth of larger trees, especially beech, was more negatively affected during post‐drought years. Therefore, an extreme drought event can indeed alter the growth partitioning within forest stands. The *DC* indicates such changes in partitioning and, hence, which trees can be selected for commercial thinning, or released from competition, to minimize potential impacts of droughts.

## Graphical abstract

The 2018 European megadrought significantly reduced the tree growth and altered the growth partitioning of European beech trees, with smaller trees showing greater growth recovery than large trees.

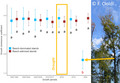

## INTRODUCTION

Tree growth responses to drought can depend on tree sizes (D'Amato *et al*. 2013; Schwarz & Bauhus 2019) and stand characteristics (Bottero *et al*. 2017; Bose *et al*. 2018; Andrews *et al*. 2020). For example, small *Pinus nigra* trees were more drought sensitive, in terms of growth, in mixed conifer stands in Spain (Martín‐Benito *et al*. 2008), while large trees were more sensitive in mixed *Picea abies* and *Fagus sylvatica* stands in Germany (Pretzsch *et al*. 2018), as well as in pure *Pinus nigra* stands in Spain (Lucas‐Borja *et al*. 2021). Several studies reported higher mortality rates of large trees under drought stress (Bennett *et al*. 2015; Olson *et al*. 2018), while others documented higher mortality of smaller trees (van Mantgem *et al*. 2009; Colangelo *et al*. 2017). These contrasting patterns emphasize the importance of identifying the individuals (size classes and species) within specific ecosystems that are most vulnerable to droughts (Dorado‐Liñán *et al*. 2019; Stovall *et al*. 2019). However, many studies focus on the largest trees (*e.g*. Hereş *et al*. 2014; Cailleret *et al*. 2017) or use non‐probabilistic samples (Peng *et al*. 2011), which means tree‐level responses cannot reliably be used to infer or predict stand‐level responses (Forrester 2019; Trugman *et al*. 2021).

Information about stand‐level responses to drought and how they relate to tree‐level responses are not only important for understanding the effects of droughts on forests but also for developing management strategies (D'Amato *et al*. 2013; Conte *et al*. 2018; Teets *et al*. 2018). By killing or weakening trees, drought significantly changes the growing environment for the surviving individuals (Bolte *et al*. 2010; Batllori *et al*. 2020), and potentially favours one group of trees over others (*e.g*. small *versus* large or shade‐intolerant *versus* shade‐tolerant) (Lloret *et al*. 2012; Grote *et al*. 2016; Pretzsch *et al*. 2018). Stand growth will be strongly reduced if the size classes that suffer also contributed a high proportion of the stand basal area growth, unless other size classes can compensate (Trouvé *et al*. 2014). Therefore, to better understand and link tree‐ and stand‐level responses it is necessary to consider (i) the tree responses in terms of differences between tree sizes or species, (ii) the tree size distributions and (iii) the stand density (Forrester 2019).

Two indices commonly used to quantify the partitioning of stand growth between trees of different sizes are the dominance coefficient (*DC*; Equation (1), see below) (Binkley 2004) and the shape or slope of relationships between tree size and tree growth (*SGR*) (Castagneri *et al*. 2012; Looney *et al*. 2018; Dye *et al*. 2019; Forrester 2019). These indices indicate which size classes (small or large) contribute more growth to the stand relative to their size (West 2014) and therefore also indicate the symmetry of competition (Pretzsch & Biber 2010; Looney *et al*. 2018; Forrester 2019). The *DC* also accounts for size distributions and has been used to plan thinning interventions or to determine whether previous thinning interventions have been successful (Soares *et al*. 2017; Forrester 2019; Lemire *et al*. 2020). Limited attention has been given to whether extreme drought events change the growth partitioning across tree size classes and species.

In 2018, large parts of Switzerland experienced a severe drought that lasted throughout the growing season (April–October) (Gharun *et al*. 2020; Schuldt *et al*. 2020). The drought was more severe in terms of growing season temperature and vapour pressure deficit than any other drought over the past 100 years and had a massive impact on forest vegetation, including widespread leaf discoloration, premature leaf shedding, xylem hydraulic failure and tree mortality (Schuldt *et al*. 2020). European beech (*Fagus sylvatica* L.) was particularly affected in terms of increased crown transparency and leaf browning, and reduced tree basal area growth and carbon sequestration (Rohner *et al*. 2021). In addition, there was a negative impact on European beech physiological (*e.g*. transpiration rate and hydraulic functions) and reproductive (*e.g*. beechnut production) (Nussbaumer *et al*. 2020; Walthert *et al*. 2021) capacity.

A special survey on selected plots of the Swiss National Forest Inventory (NFI), which includes measurements of the same plots before and after the 2018 drought *(*Rohner *et al*. 2021), provided an opportunity to examine whether this extreme event influenced growth partitioning. A potential methodological limitation of using these NFI plots for calculating dominance coefficients is the small number of trees per plot due to the plot size (500 m^2^). There has been very little work to determine minimum sample sizes (trees per plot) required to calculate growth partitioning indices (Ducey 2010). Given the growing interest in growth partitioning within forests (*e.g*. Bradford *et al*. 2010; Trouvé *et al*. 2014; Baret *et al*. 2017; Pothier 2017; Fernández‐Tschieder *et al*. 2020), and the abundance of NFI data, especially across different countries in Europe, there is an urgent need to examine how the values of growth dominance indices might be influenced by small sample sizes, stand structures (even‐aged *versus* single‐tree selection forests) and the range of tree sizes within a sample.

In this study, we examined whether the 2018 drought shifted the partitioning of growth among tree sizes and species. Specifically, we asked (i) did growth partitioning differ before, during and after the 2018 drought, and, if so, did that change occur equally in beech‐dominated and beech‐admixed plots; (ii) did the relationship between tree growth and tree size or stand basal area differ prior to and after the 2018 drought; and (iii) did site and climatic factors, including soil moisture and air temperature, influence the growth partitioning in beech‐dominated and beech‐admixed plots? To do so, we first examined the influence of sample size on growth dominance indices (*DC* and *SGR*) and whether this depends on stand structure (*i.e*. even‐aged *versus* single‐tree selection forests) and ranges of tree sizes within the sample.

## METHODS

### Role of sample size on growth dominance indices

For quantifying the role of sample size on *DC* and *SGR* calculations, we used plots from the Experimental Forest Management (EFM) network of Switzerland (Forrester *et al*. 2019), which typically has a larger range and mean of plot sizes and tree numbers per plot than the NFI plots. The EFM plots also have more accurate records of which trees were thinned or died. Relative to the NFI plots used in this study, the EFM plots cover a wider range of forest structures, species compositions and management types. This coverage enlarges the scope of our analysis and makes the results more widely applicable. The EFM network is used to examine the effects of silvicultural treatments across a range of species, climate and edaphic conditions. In this study we selected EFM plots with at least 100 trees; tree growth was inventoried at least twice and characterized by one of the two following stand structures: even‐aged (unimodal diameter distributions) or single‐tree selection stands (negative exponential shaped diameter distributions). We considered these two stand structures because they represent two extreme types of tree‐size distributions. This resulted in 868 EFM plots with varying tree growth measurement years between 1888 and 2020. Plot sizes ranged from 0.037 ha to 11.0 ha, with a mean 0.36 ha (Forrester *et al*. 2019).

For each tree in EFM plots, the diameter at 1.3 m height (*d*), status (live, dead, thinned) and species were recorded. The growth between successive inventories was calculated in terms of increment in *d* (cm), basal area (m^2^) and stem mass (kg). Stem mass was calculated for each measured tree using equations developed for European forests (Forrester *et al*. 2017). For quantifying the role of sample size on growth partitioning, we considered two indices: (i) the growth dominance coefficient (*DC*; Binkley 2004) and (ii) size–growth relationship (*SGR*; Metsaranta & Lieffers 2010; Castagneri *et al*. 2012).

The *DC* was quantified according to West (2014), by arranging the trees in ascending order of size (*d*, basal area or stem mass), calculating the cumulative proportion of stand sum of *d*, basal area or stem mass contributed by each tree, then plotting that against the cumulative proportion of stand growth (in terms of *d*, basal area or stem mass) contributed by each tree:

(1) $$DC=1‐\sum_{i=1}^{n}\left(s_{i}‐s_{i‐1}\right)\left(\Delta_{i}+\Delta_{i‐1}\right)$$

where *s_i_* is the cumulative proportional size of tree *i*, and Δ*_i_* is the cumulative proportional growth of tree *i*. The *DC* varies between −1 and 1, where *DC* = 0 indicates that trees grew in proportion to their size, *DC* > 0 indicates that large trees grew disproportionately more for their size, and *DC* < 0 indicates that small trees grew disproportionately more for their size (Binkley 2004; Binkley *et al*. 2006).

The *SGR* was quantified as the slope of a linear relationship between proportional stand growth (*e.g*. individual growth relative to stand growth) and proportional size (*i.e*. individual size relative to the sum of all trees in the stand) (Metsaranta & Lieffers 2010; Castagneri *et al*. 2012; Looney *et al*. 2018). As for the *DC*, we calculated *SGR* using individual‐level tree growth in terms of *d*, basal area and stem mass.

For quantifying the role of sample size on growth partitioning, we first calculated the *DC* and *SGR* using all trees within the plot. We then calculated the *DC* and *SGR* for the same plots but using sample sizes of 5, 8, 10, 20, 30, 50 and 100 trees to match the range of sample size of the NFI plots, which were randomly selected from the plots. We then quantified the Pearson correlation coefficient of the relationship between a partitioning index calculated for all trees in the plot and the same index calculated using the smaller sample sizes.

To examine the possible effects of particularly large trees on *DC*, we compared the *DC* calculated using 100 trees randomly selected within a plot, with the *DC* calculated after removing the largest ten and 20 trees. This comparison was done based on pairwise Wilcoxon signed rank tests.

### Effects of the 2018 drought on growth partitioning

**Fig. 1:**
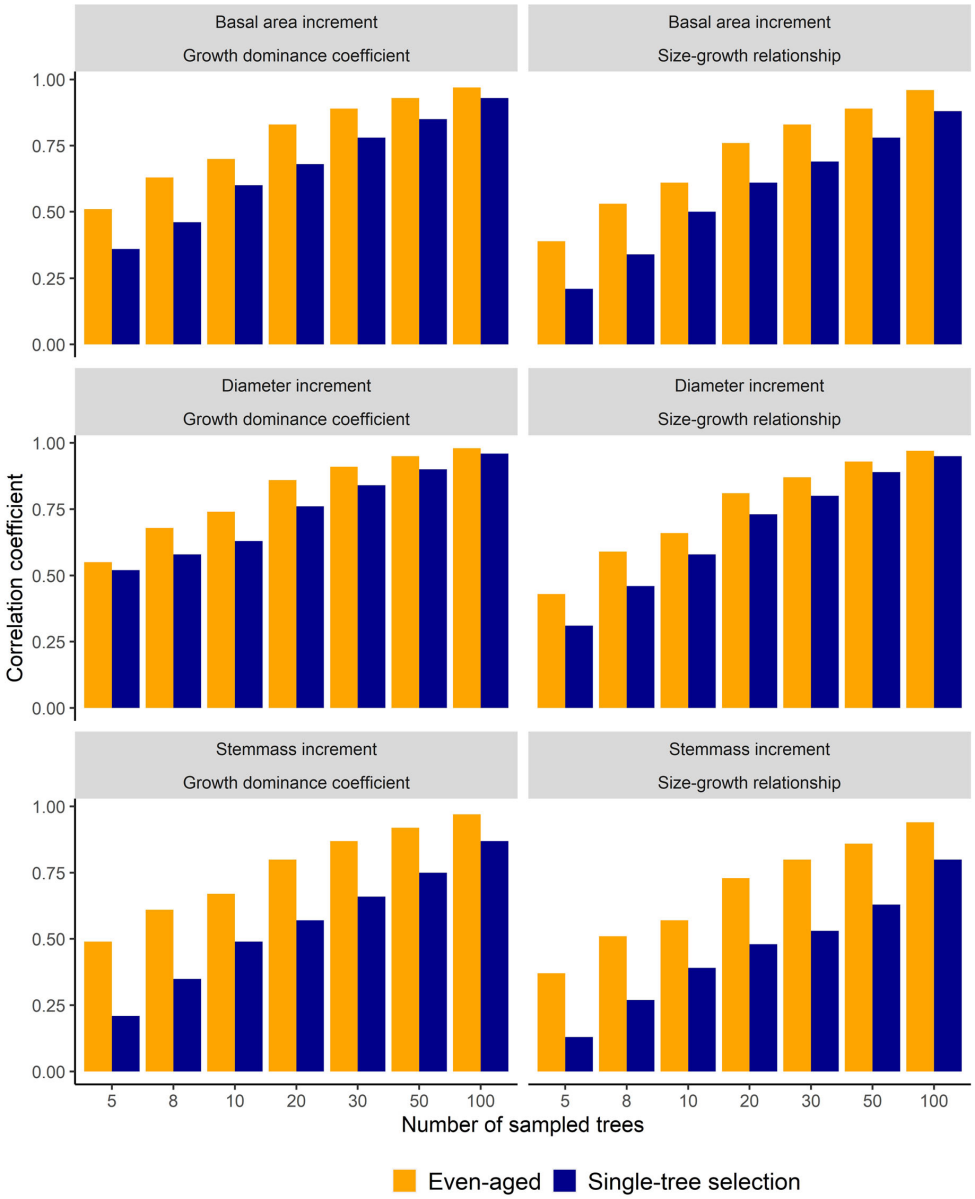
Pearson correlation coefficient or relationships between growth dominance coefficient (*DC;* left panels) or size‐growth relationship (*SGR*; right panels) calculated from all trees within a plot compared with the same coefficients calculated with a different number of randomly selected sample sizes. The analyses were performed for even‐aged and single‐tree selection stands. Individual tree increment was quantified from tree DBH, basal area, and stem mass.

To examine the effect of the 2018 drought (Figure S1) on growth partitioning, we used NFI plots. Typically, these plots are remeasured at approximately 10‐year intervals. However, a subset of 75 plots was measured more frequently specifically to examine the effects of the 2018 drought (Rohner *et al*. 2021). We selected plots with at least eight trees, because our examination of the effect of sample size on *DC* revealed correlation coefficients < 0.50 for sample sizes lower than eight trees (Fig. 1; see Vigiak *et al*. (2018) and Wu *et al*. (2013) for the use of 0.50 as threshold in ecological studies).

Four inventory measurements were conducted prior to the 2018 drought, including measurements in 1983–1985, 1993–1995, 2004–2006, 2009–2010, and in spring 2018. We assumed that the last growing season represented by the measurement in spring 2018 is 2017, *i.e*. that the spring measurement has taken place before the substantial onset of the growing season. Three inventory measurements were conducted during and after the 2018 drought in the months of August and September 2018, 2019 and 2020 (Rohner *et al*. 2021). For consistency, we characterized the growth periods of all measurements mentioned above as pre‐2018 drought periods: 1984–1994, 1995–2004, 2005–2009, 2010–2017, 2018‐drought period: 2018, and post‐2018 drought periods: 2019 and 2020 (Table 1).

**Table 1:** Number of Swiss National Forest Inventory (NFI) plots used in the analysis for each measurement period.

Measurement periods	Beech‐dominated stands	Beech‐admixed stands
1984–1994	6	16
1995–2004	8	20
2005–2009	9	31
2010–2017	13	35
2018 (drought year)	12	36
2019	12	43
2020	12	42

The Swiss NFI plots are circular nested plots, where every tree with *d* ≥ 12 cm is recorded within an inner 200‐m^2^ circle (horizontal radius = 7.98 m), and every tree with *d* ≥ 36 cm is recorded within a 500‐m^2^ circle (horizontal radius = 12.62 m). The basal area and basal area increment were calculated for each tree and used to calculate the *DC* for each growth period. Following the results of the first part of the data analyses, we did not use NFI data to calculate *SGR*, and *DC* was only calculated using tree basal area and not tree diameter or stem biomass. We annualized the growth data (*i.e*. basal area increment per year) when the measurement interval was longer than 1 year.

The *DC* was analysed in relation to growth periods (seven levels: 1984–1994, 1995–2004, 2005–2009, 2010–2017, 2018, 2019 and 2020), forest stand type: beech‐dominated stands (*i.e*. ≥ 50% of stand basal area was beech) and beech‐admixed stands (*i.e*. <50% of stand basal area was beech), and the interactions between measurement period and forest stand type. The percentage of total stand basal area contributed by beech in beech‐dominated and beech‐admixed plots was 70% and 15%, respectively (Table S1). We considered the measurement period 2017–2018 (*i.e*. drought period) as reference in the linear‐mixed effect model (Zuur *et al*. 2009). We performed the *post‐hoc* Tukey multiple comparison test to detect statistical differences (Hothorn *et al*. 2008) using the *lsmeans* package in R (Lenth 2016). The repeated measurements associated with each plot were treated as a random effect variable. The linear mixed‐effect modelling was executed using the *lme* function of the *nlme* package in R (Pinheiro *et al*. 2014; R Development Core Team 2018).

### Effects of the 2018 drought on annual basal area increment

The effect of the 2018 drought on tree‐level annual basal area increment (BAI) was examined by modelling tree‐level annual BAI as a function of the growth periods (seven levels: 1984–1994, 1995–2004, 2005–2009, 2010–2017, 2018, 2019 and 2020), forest stand types (two levels: beech‐dominated stands and beech‐admixed stands), and the interactions between growth period and forest stand type. We performed linear mixed‐effect modelling for this analysis, where trees nested within plots were treated as a random effect variable.

The effect of tree size (*i.e*. tree basal area) and of stand basal area on annual BAI were quantified as the slope of the linear‐mixed effect model, where annual BAI was modelled as a function of tree size and stand basal area. We quantified the effect of tree size and of stand basal area on annual BAI for each measurement period and for each forest type.

### Effects of site and climatic factors on growth partitioning

We also examined the effects of soil characteristics (pH of the upper soil and soil water‐holding capacity at 1 m soil depth) and topography (elevation and slope) on *DC*. These variables were obtained from Remund *et al*. (2014) and Abegg *et al*. (2014) as reported in Rohner *et al*. (2018). Site‐specific climate data were obtained from MeteoSwiss for the period from 1981 to 2020. Monthly precipitation sums (mm), monthly mean air temperatures (°C) and monthly solar radiation (W m^−2^) from January to December were obtained. Seasonal temperature and precipitation variables were computed by averaging monthly values (summer: June–August; autumn: September–November; winter: December–February; spring: March–May). Monthly potential evapotranspiration was calculated using the *Thornthwaite* function of the R package SPEI (Begueria & Vicente‐Serrano 2013).

The *DC* was analysed in relation to site variables (soil pH, soil water‐holding capacity, site elevation and slope) and climate variables (seasonal‐ and annual‐scale temperature, precipitation, potential evapotranspiration and solar radiation, averaged for the period 1981 to 2018). We developed several linear mixed‐effect models, and the top‐ranked model for *DC* was obtained by comparing the Akaike Information Criterion (AIC) weight with the full model. The full model incorporated the effects of all predictor variables mentioned earlier. However, in the full model, we excluded highly correlated variables (*i.e*. correlation coefficient >0.50). For this analysis, the repeated measurements associated with each plot were treated as a random effect variable.

## RESULTS

### Role of sample size on growth dominance indices

We found a strong effect of sample size on both *DC* and *SGR*. The correlation coefficient for both *DC* and *SGR* decreased with smaller sample sizes (Fig. 1). The magnitude of the decrease in correlation coefficient was stronger for the *SGR* than the *DC*. Sample size was more important in single‐tree selection stands than even‐aged stands. In addition, increment in terms of stem mass was more sensitive to sample size compared to increment in terms of diameter or basal area. Our results showed that the correlation between *DC* based on large sample sizes (n > 100) and smaller samples sizes declined from 0.90 (n = 50) to 0.85 (n = 20), 0.70 (n = 10), 0.65 (n = 8) and 0.5 (n = 5) in even‐aged forests and to 0.80, 0.75, 0.65, 0.45 and 0.35 in single‐tree selection forests (Fig. 1). *SGR* required larger sample sizes and was not used to examine drought responses. For the rest of the analyses, we therefore only included *DC* calculated using sample sizes ≥ 8 trees.

**Fig. 2:**
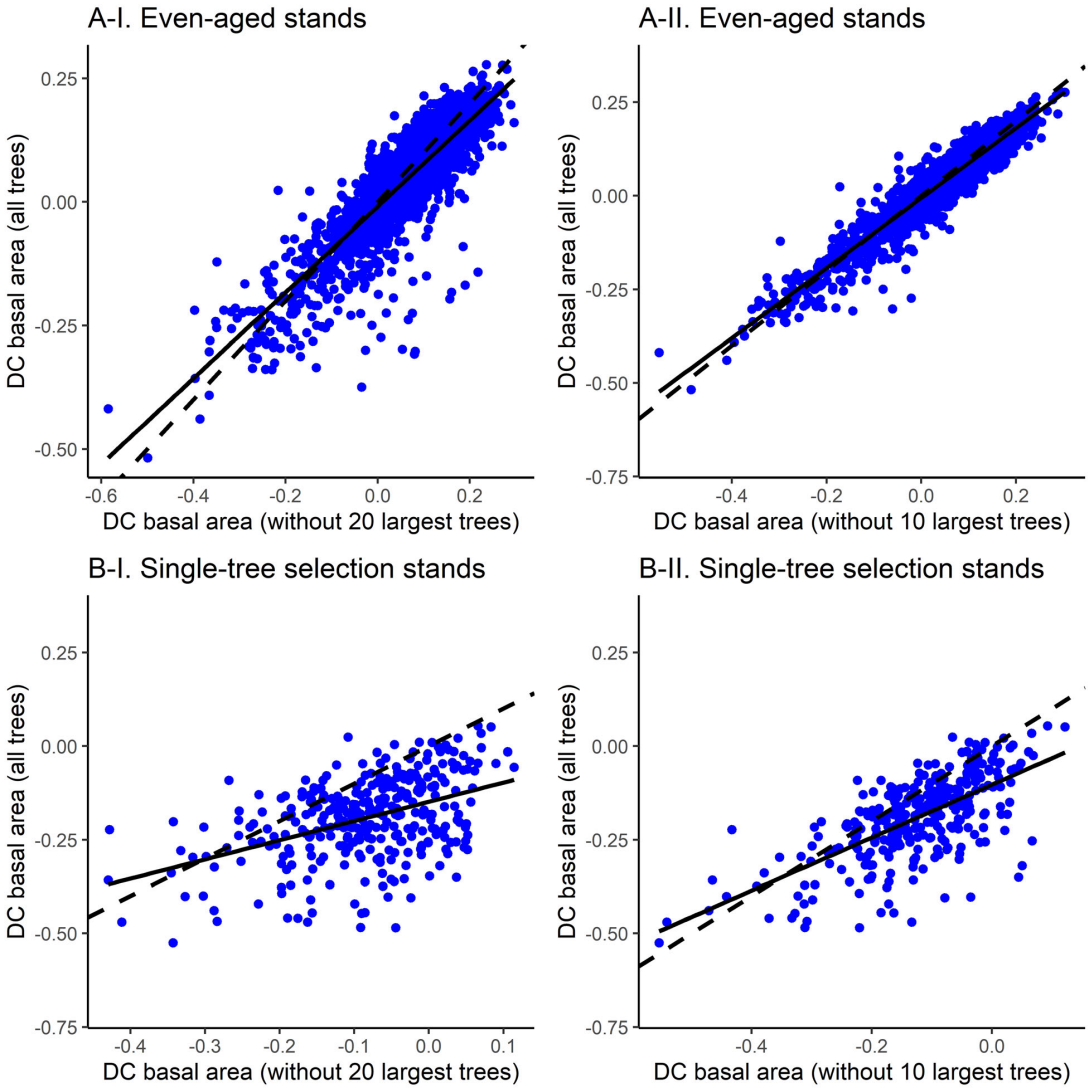
The role of the presence of the largest trees in quantification of the growth dominance coefficient (DC) in even‐aged and single‐tree selection stands. Solid line represents the fitted line of the linear model while dashed line represents the 1:1 line with intercept 0 and slope 1. All pairwise Wilcoxon signed rank tests between DC calculated from all trees and after removing the largest trees were significant (*P* < 0.0001).

Our results showed that samples that do not cover the whole tree size range can lead to biased *DC* values. The pairwise Wilcoxon signed rank tests revealed that *DC* calculated from 100 randomly sampled trees per plot significantly differed from *DC* calculated after removing the largest ten and 20 trees from these samples (Fig. 2). The role of tree size range was stronger in single‐tree selection stands compared to even‐aged stands (Fig. 2).

### Effects of the 2018 drought on growth dominance coefficient

**Fig. 3:**
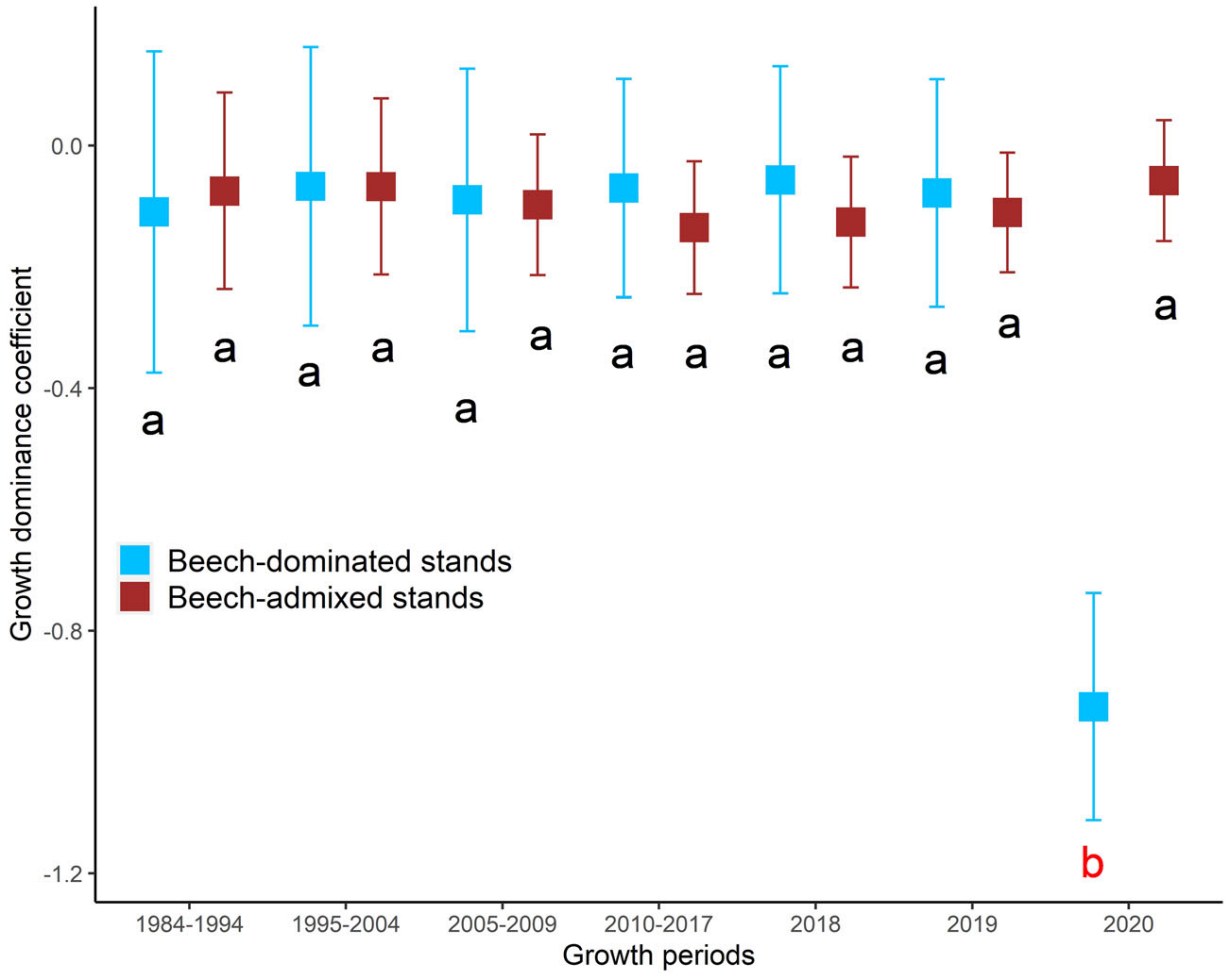
Growth dominance coefficient of beech‐dominated and beech admixed stands over a 36‐year monitoring period incorporating periods before and after the 2018 drought. The growth in 2018 (*i.e*. drought period) was considered as the reference in the linear model. Letters on top of each bar show the results of posthoc Tukey multiple comparison test.

The *DC* values were consistently lower than zero over the 36‐year monitoring period, irrespective of forest stand types or the growth period (Fig. 3). We found no differences in *DC* prior to the 2018 drought and during the drought for the whole stand. However, when considering only the *DC* of beech‐dominated stands, *DC* was lower during the post‐drought year compared to during the drought or any other growth periods that we examined in this analysis (Fig. 3).

### Effects of the 2018 drought on annual basal area increment

**Fig. 4:**
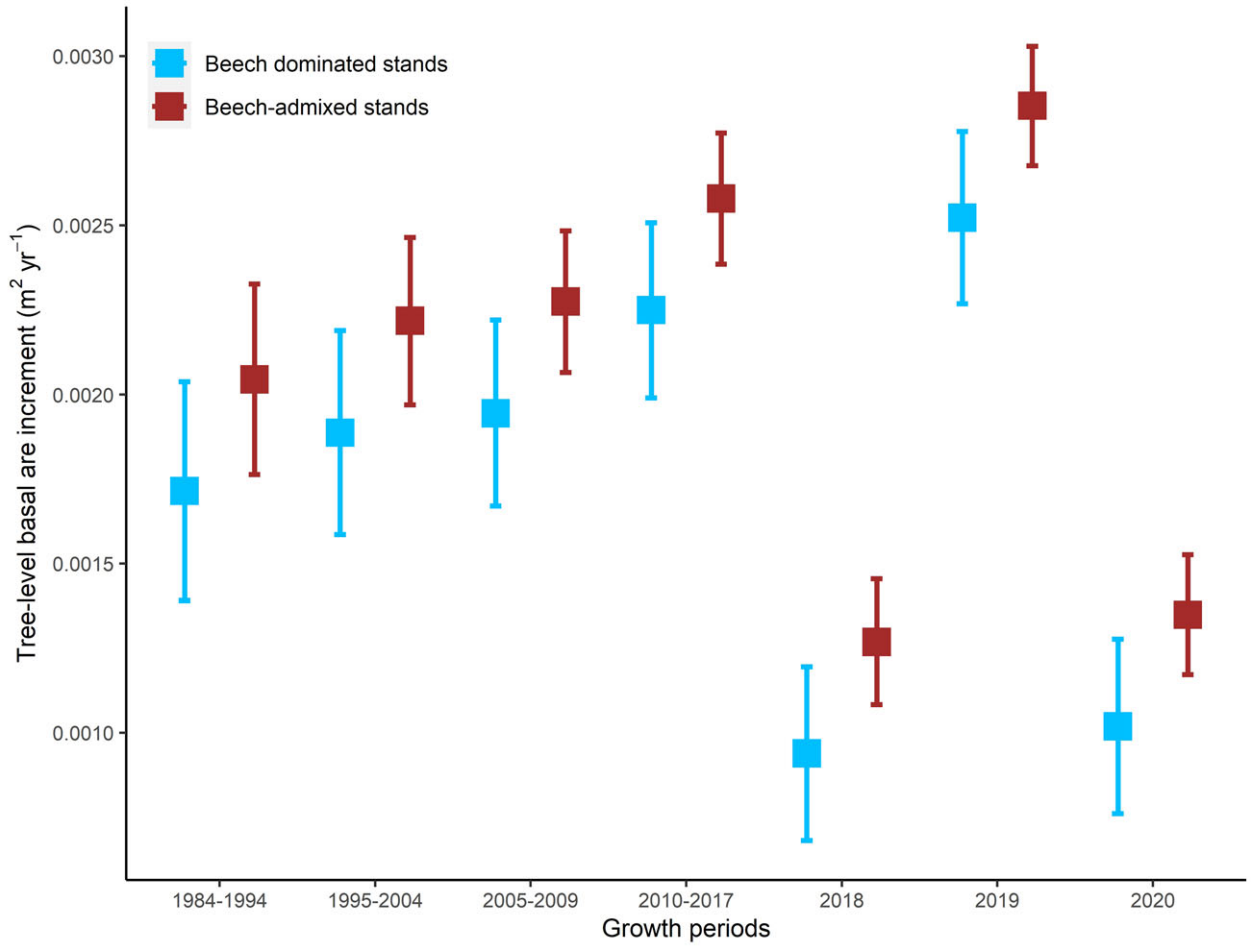
Annual tree‐level basal area increments (BAI) of beech‐dominated and beech‐admixed stands over a 36‐year monitoring period incorporating periods before and after the 2018 drought. The growth in 2018 (*i.e*. drought period) was considered as the reference in the linear‐mixed effect model. BAI was significantly lower during the 2018‐drought period compared to other periods except the BAI in 2020. No statistically significant difference was observed between the two stand types.

The annual BAI was significantly lower in 2018 (*i.e*. period of 2018 drought) compared to all other periods, except 2020 (Fig. 4). After the 2018 drought, trees recovered their growth during the following year (*i.e*. 2019), but did not maintain the annual BAI of 2019 in 2020 (Fig. 4). The differences between beech‐dominated and beech‐admixed stands were not significant (Fig. 4).

**Fig. 5:**
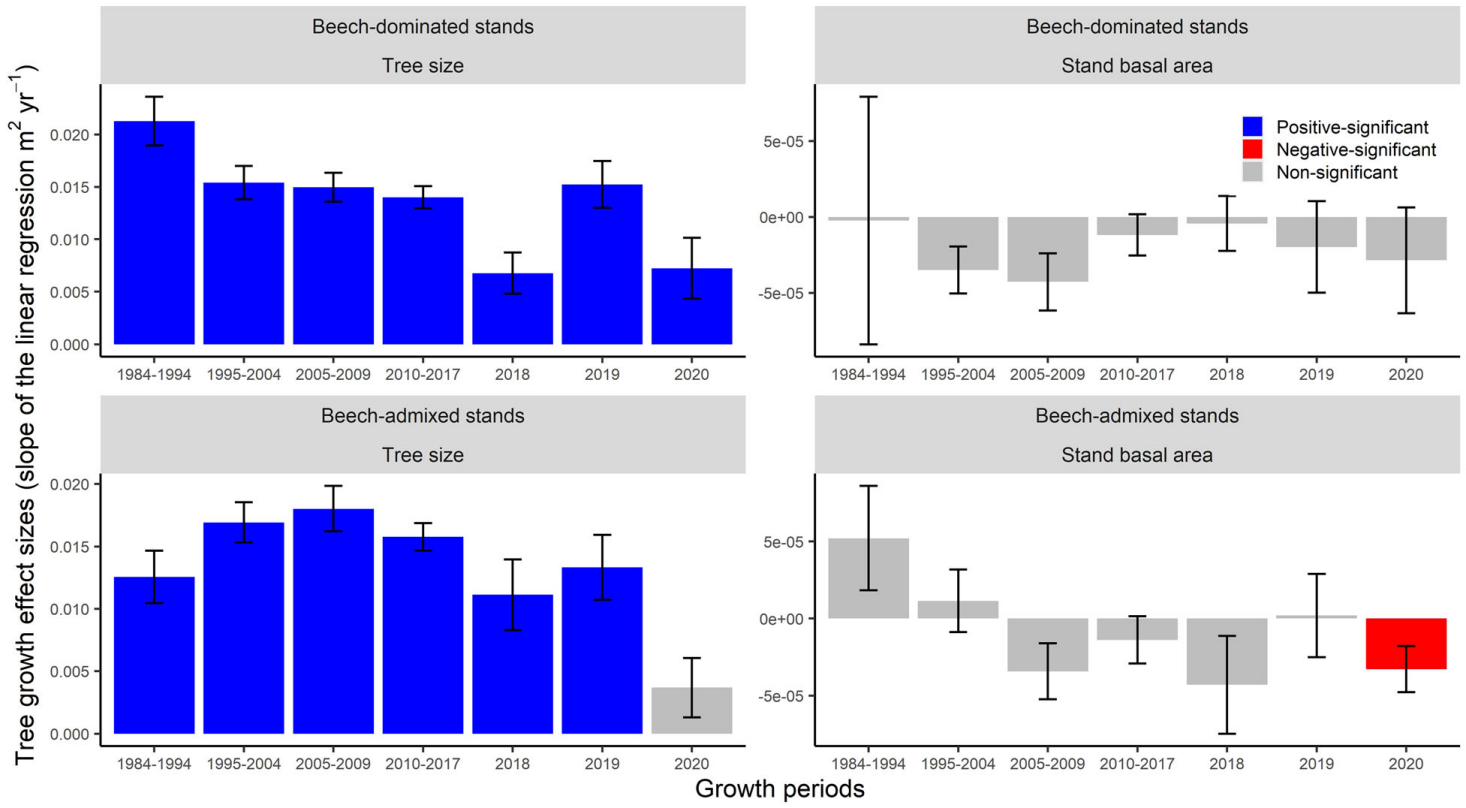
Effect size of tree size and stand basal area determining the tree‐level annual basal area increment of beech‐dominated and beech‐admixed stands over a 36‐year monitoring period incorporating periods before and after the 2018 drought. The effect is measured by the slope of the linear mixed‐effect model. The error bars represent the mean ± standard errors.

**Fig. 6:**
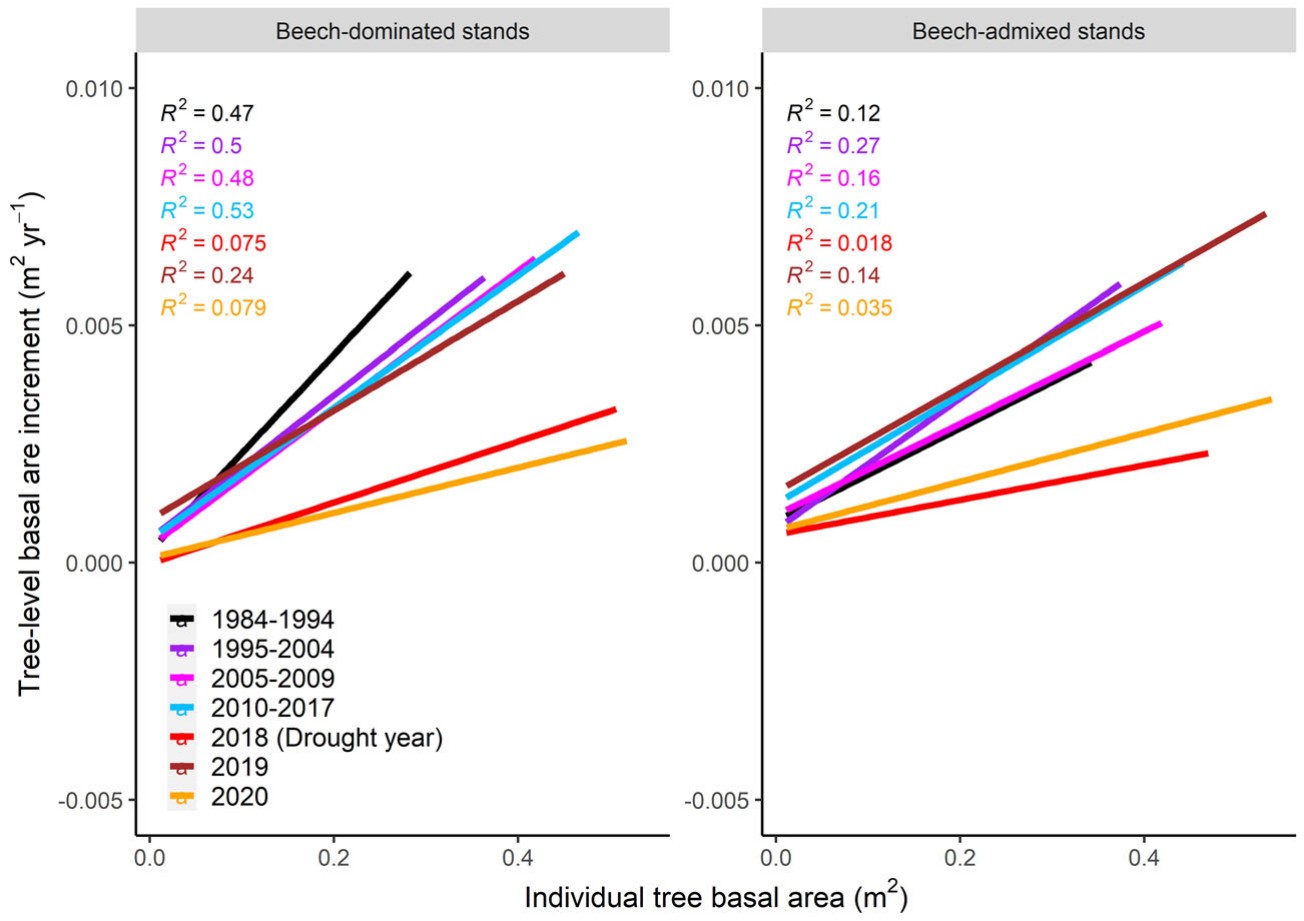
Fitted linear regression lines and associated R^2^ for the relationship between individual tree size (basal area, m^2^) and tree‐level annual basal area increment (BAI, m^2^ yr^‐1^) of beech‐dominated and beech‐admixed stands over a 36‐year monitoring period incorporating periods before and after the 2018 drought. For all periods, the relationship between individual tree size and tree‐level annual BAI was significant (*P* < 0.05) except 2020 in beech‐admixed stands (see Fig. 5).

Tree annual BAI was positively related to tree size across all growth periods, except the tree annual BAI in 2020 in beech‐admixed stands (Fig. 5). The effect size of tree basal area on annual BAI decreased during the 2018 drought as well as during the two post‐2018 drought years, as indicated by the slope of the linear regression (Fig. 5) and visualized in Fig. 6. Stand basal area had a negative effect on tree annual BAI for 2020 in beech‐admixed stands, but not for any other periods or stands (Fig. 6).

### Effects of site and climatic factors on growth dominance coefficient

Our analysis detected a significant positive relationship between soil water‐holding capacity and *DC*. However, this significant relationship was only visible in the top‐ranked model (*i.e*. model with the lowest AIC value) but not in the full model. We detected no significant relationship between *DC* and any other soil, site or climate‐related variables considered in this analysis irrespective of seasonal or annual resolution (Table 2).

**Table 2:** Linear mixed‐effect model: fit of growth dominance coefficient as a function of different geographic and climate variables incorporated in the best model and in the full model.

Model components	Best model Estimate (±SE)	Full model Estimate (±SE)
Intercept	0.514 (0.604)	−2.115 (5.497)
Soil water‐holding capacity (mm)	0.003 (0.001)*	0.001 (0.001)
Soil pH	—	0.022 (0.027)
Slope of the site	—	−0.002 (0.003)
Mean annual temperature (°C)	−0.074 (0.047)	−0.254 (0.189)
Spring precipitation sum (mm)	0.002 (0.001)	−0.001 (0.002)
Summer potential evapotranspiration (mm)	—	0.015 (0.016)
Growing season solar radiation (W m^2^)	—	−0.0001 (0.0001)
AIC_c_	623.3	667.7
$R_{m}^{2}$	0.02	0.03
$R_{c}^{2}$	0.02	0.03

The model comparison was based on AIC_c_ (Akaike’s Information Criterion). $R_{m}^{2}$ = marginal R^2^ (variance explained by the fixed factors); $R_{c}^{2}$ = conditional R^2^ (variance explained by the fixed and random factors). Significance: *P< 0.05. Climate data (*i.e*. mean annual temperature, spring (March–May) precipitation sum, growing season (March–August) solar radiation and summer (June–August) potential evapotranspiration) are the averages of data for the years 1981 to 2018.

## DISCUSSION

### Sample size sensitivity of growth dominance coefficient

Our results showed that the precision of *DC* declines as sample size declines, and this effect is stronger for single‐tree selection forests (negative exponential‐shaped *d* distributions) than for even‐aged forests (uni‐modal *d* distributions). Therefore, the suitability of datasets to calculate *DC* depends not only on the tree sample size, but also on the shape of the *d* distributions.

The *DC* also depended on the range of tree sizes. It is worth noting that the analysis on the effect of the size range was unlikely to be impaired by the sample size reduction from 100 to 90 and 80 trees when removing the largest trees per plot, as reducing the sample size from 100 to 50 trees affected DC much less than further sample size reductions (Fig. 1). The identified dependence on the presence of the largest trees means treatments that differ in size ranges (*e.g*. different stand age) or treatments that directly change the size range (*e.g*. thinning) can cause differences in *DC* even if the size–growth relationships are the same. The reason for this is that when the size–growth relationship is not linear (or is linear, but does not pass through the origin), the removal of trees at one end of the size range will remove trees that have a higher (or lower) growth per size than other trees, which will decrease (or increase) the *DC*. Many studies have used the *DC* to examine how thinning has influenced growth partitioning (Bradford *et al*. 2010; Trouvé *et al*. 2014; Forrester 2019; Lemire *et al*. 2020). This study indicates that if thinning modifies the size range, part of the effects will be due to the change in size range, in addition to any changes in size–growth relationships or size distributions (Forrester 2019).

### Effects of the 2018 drought on tree growth and growth partitioning

Our study identified changes in growth partitioning due to the 2018 drought, which supports the argument that tree size and stand characteristics modify the drought impact in forest stands (D'Amato *et al*. 2013; Pretzsch *et al*. 2018; Andrews *et al*. 2020; Bottero *et al*. 2021). Although the 2018 drought did not change the *DC* during the drought period, the *DC* in beech‐dominated stands was negatively affected during the second year after the 2018 drought (Fig. 3). During this growth period (*i.e*. second year after drought), smaller trees may have been partly released from competition from larger trees. The larger negative impact of drought on larger trees could be due to increased exposure to solar radiation and higher evaporative demand (Nepstad *et al*. 2007; Baret *et al*. 2018). Larger trees can be physiologically maladapted to extreme drought events (Bennett *et al*. 2015). Tree height is often positively related to mortality risk (Stovall *et al*. 2019), because tall trees have to lift water to greater heights against the effect of gravity and may face increased hydraulic challenges (Olson *et al*. 2018). In addition, larger trees with crowns directly exposed to increased solar radiation and higher vapour pressure deficit during drought can struggle to stay within a safe hydraulic margin while maintaining open stomata for C assimilation (Baret *et al*. 2018; Choat *et al*. 2018).

The negative *DC* values that we found in these stands during droughts and post‐drought years indicated that the growth of dominant trees is lower than their proportional contribution to stand basal area (Binkley *et al*. 2006), indicating the importance of small trees to whole stand growth. Drought may create more favourable conditions for smaller understorey trees by causing defoliation of taller overstorey beech trees (Rohner *et al*. 2021; Walthert *et al*. 2021), allowing an increased penetration of solar radiation to the shorter trees. Therefore, trees with inferior social positions (*i.e*. shaded) may benefit from the reduced water consumption of tall canopy trees and from being shaded in dry years (Goisser *et al*. 2016; Pretzsch *et al*. 2018). The growth reduction of larger trees due to drought was made up for by the smaller trees, partly because smaller trees were less affected by drought but also because they contributed a large proportion of stand density (negative exponential *d* distributions) and growth. A similar finding, *i.e*. growth partitioning changes in favour of small trees under dry conditions in beech‐dominated stands, was reported by Pretzsch *et al*. (2018). In contrast, the opposite was found for Douglas‐fir (*Pseudotsuga menziesii*)‐dominated stands, where growth partitioning shifted in favour of large trees under dry conditions (Trouvé *et al*. 2014).

The species‐specific response pattern might be explained by the anisohydric trait of European beech, which is vulnerable to long‐lasting severe droughts (Leuschner *et al*. 2021) such as the 2018 drought that lasted the entire growing season (Schuldt *et al*. 2020). The anisohydric trait can cause vulnerability in trees to extreme droughts as they have weaker control on stomata and continue to transpire at high levels of embolism (McDowell *et al*. 2008; Hartmann 2011; Walthert *et al*. 2021). The 2018 megadrought caused significant physiological damage to beech trees and resulted in substantial crown defoliation (Walthert *et al*. 2021). Our study showed that the growth of the large beech trees was more negatively affected by the 2018 drought compared to the small trees.

Prior to the 2018 drought, tree basal area growth was strongly related to tree size (Figs 5 and 6). However, that positive linear relationship was weakened (reduced slope and R^2^) during the drought and was not even significant for the second year after the drought in beech‐admixed stands (Fig. 5). These results clearly show that larger trees were more affected by the drought, and that the impact of drought was still significant 2 years after the drought occurred. However, why the growth reduction occurred only in the second year after the drought—but not the first—remains unclear and needs further investigation. These carryover or legacy effects of drought during the post‐drought years have also been reported in other studies (Anderegg *et al*. 2015; Peltier & Ogle 2019; Kannenberg *et al*. 2020; Bose *et al*. 2021). Severe droughts can cause structural and physiological damage to trees and can activate repair mechanisms that eventually lead to reduced tree radial growth during post‐drought years (Ovenden *et al*. 2021). However, the 2018 megadrought did not significantly alter the growth partitioning in beech‐admixed stands (Fig. 3), which may indicate the higher resilience of mixed‐species forests to extreme droughts, and support the arguments of many studies that promote mixed‐species forest compositions (Bauhus *et al*. 2017; Bottero *et al*. 2021; Pardos *et al*. 2021).

### Factors affecting the growth dominance coefficient

The significant positive relationship between soil water‐holding capacity and *DC* indicates that large trees contribute more to stand growth under moist conditions. This may result from several size‐dependent tree interactions that facilitate the resource uptake and resource‐use efficiency of taller trees relatively more than smaller trees, *e.g*. that taller trees have better access to light than smaller trees (Forrester 2019). However, growth partitioning shifted in favour of smaller trees under drought conditions. There are several size‐dependent tree interactions that can favour smaller trees (Forrester 2019). For example, larger trees carry more photosynthetic biomass and require a higher water availability for maintaining photosynthetic activity for growth and defence (Ryan & Yoder 1997; Scholz *et al*. 2011), which can make them vulnerable under drought conditions (Olson *et al*. 2018; Stovall *et al*. 2019). However, it is important to mention that we observed non‐significant relationships between *DC* and precipitation and/or potential evapotranspiration. In addition, the differences in measurement intervals between successive inventories did not allow us to associate the temporal soil water data with temporal *DC* measurements. This limits our ability to examine the potential role of soil water in determining the growth partitioning during drought and post‐drought years (Walthert *et al*. 2021).

In conclusion, the reliability of *DC* and *SGR* calculations declines as sample sizes decrease, and this decline appears to be greater for *SGR*. *DC* calculated from samples sizes using n = 8 trees was still highly correlated with actual *DC* (correlation coefficient > 0.5; see Wu *et al*. 2013; Vigiak *et al*. 2018). The *DC* also depends on the range of tree sizes, which means treatments that differ in size ranges or treatments that directly change the size range (*e.g*. thinning) can cause different *DC*, even if the size–growth relationships are the same. By calculating *DC* for Swiss NFI data, we found that the 2018 megadrought not only reduced tree growth but also redistributed the total stand growth in favour of smaller trees. This applied especially for European beech‐dominated stands. A practical implication is that thinning interventions selectively targeting larger trees could not only reduce competition for residual trees but also increase resistance to upcoming severe drought events. The tree size‐dependent responses to drought indicate that analyses of stand‐level growth responses to drought need to consider all tree sizes, with the possible exception of sizes that contribute only a very minor proportion of the stand density or growth.

## Supporting information

Supplementary Material
