# Supplementary material for: Did the 2018 megadrought change the partitioning of growth between tree sizes and species? A Swiss case‐study
Source: Plant Biol (Stuttg). 2021 Dec 22;24(7):1146–56. doi: 10.1111/plb.13380 (PMC10078792; doi:10.1111/plb.13380)
Supplement: Supplementary file 1 — Supplementary Material [file PLB-24-1146-s001.docx]

**Supplementary information**

**Title: Did the 2018 megadrought change the partitioning of growth between tree sizes and species? A Swiss case-study**

Author names and affiliations

Arun K. Bose^1, 2, *^, Brigitte Rohner^1,3^, Alessandra Bottero^1,3,4,5^, Marco Ferretti^1,3^, David I. Forrester^1,3^

^1^Swiss Federal Institute for Forest, Snow and Landscape Research WSL, Zürcherstrasse 111, CH-8903 Birmensdorf, Switzerland

^2^Forestry and Wood Technology Discipline, Khulna University, Khulna 9208, Bangladesh.

^3^SwissForestLab, CH-8903 Birmensdorf, Switzerland.

^4^WSL Institute for Snow and Avalanche Research SLF, CH-7260 Davos Dorf, Switzerland.

^5^Climate Change, Extremes and Natural Hazards in Alpine Regions Research Center CERC, CH-7260 Davos Dorf, Switzerland.

*Corresponding author**:** Swiss Federal Institute for Forest, Snow and Landscape Research WSL, Zürcherstrasse 111, CH-8903 Birmensdorf, Switzerland, Email: [arun.bose@wsl.ch](mailto:arun.bose@wsl.ch).

**Table SM1.** Descriptive statistics of beech-dominated and beech-admixed plots used in this study.

| **Characteristics** | **Beech-dominated plots (Mean±SD)** | **Beech-admixed plots**  **(Mean±SD)** |
| --- | --- | --- |
| Tree DBH (cm) | 35.4±8.2 | 33.2±8.4 |
| Stand density (stems ha^-1^) | 349.7±164.3 | 394.5±158.7 |
| Stand basal area (m^2^ ha^-1^) | 29.9±13.2 | 31.1±16.4 |
| Basal area of beech (m^2^ ha^-1^) | 20.8±10.5 | 4.6±4.5 |
| Basal area of Norway spruce (m^2^ ha^-1^) | 2.2±3.8 | 11.3±13.9 |
| Basal area of silver fir (m^2^ ha^-1^) | 1.7±3.1 | 6.7±11.5 |


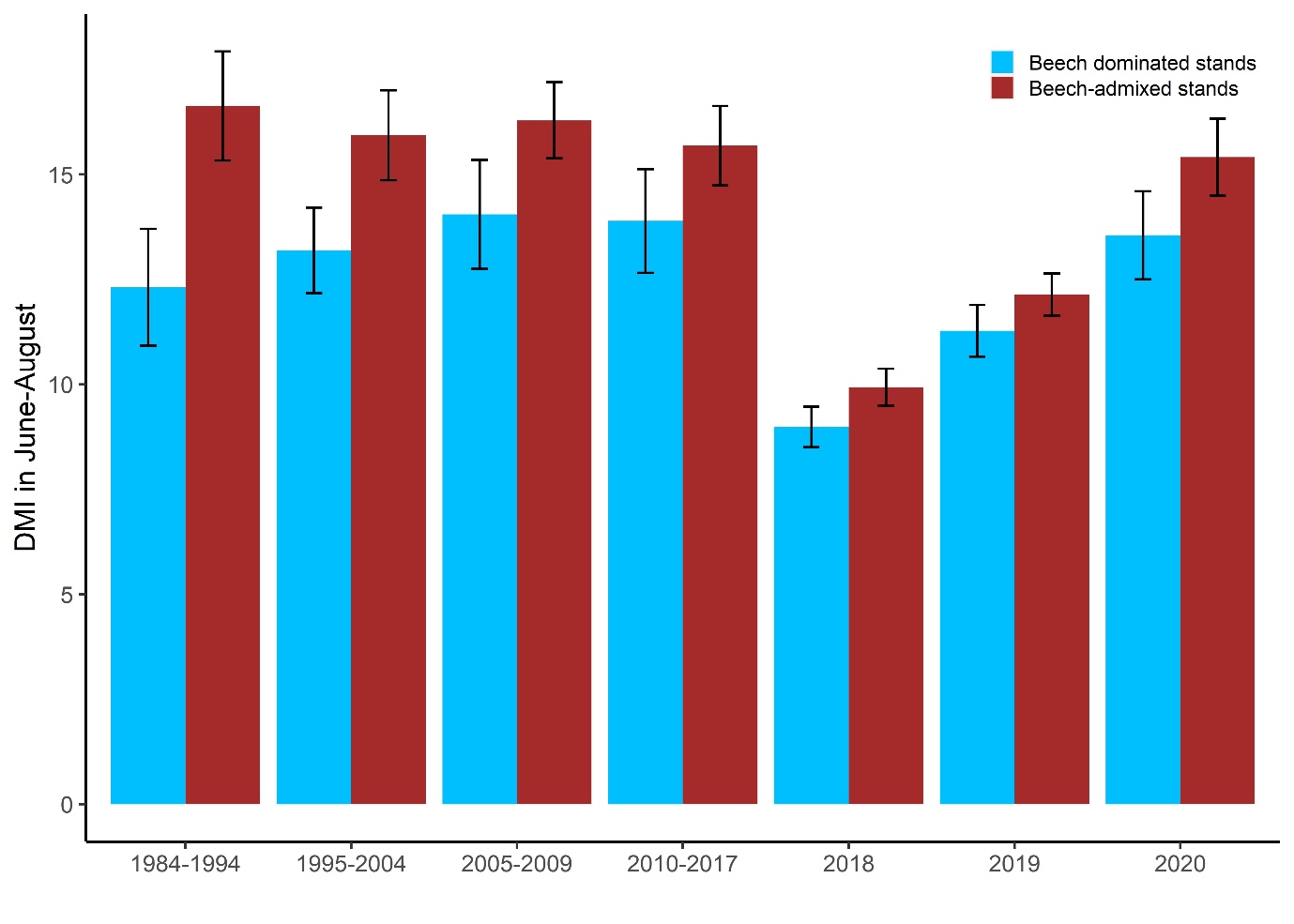


**Figure SM1.** Temporal distribution of DMI (De Martonne Index) aridity index of June-August. The DMI was calculated according to De Martonne (1926).

**Reference**

De Martonne, E. (1926). Une nouvelle fanction climatologique. L’indice d’aridite La Meteriologie, 449–458.
